# 

**Published:** 1898-01

**Authors:** 


### CONTENTS.

ORIGINAL ARTICLES: PAGE

Methods of Meat-inspection. By LEONARD PEARSON, B.S., V.M.D. . . . . 1
Some of the More Prevalent Diseases Affecting Animals. By A. W. CLEMENT, V.S. 6
Some Dangers of Milk. By T. B. POTE, D V.S. . . . . . . . . . 12
Tuberculosis and its Relation to the Veterinarian. By C. W. FISHER . . . . 16
The Close Relationship Existing Between the Veterinary Profession of To-day and Organized Societies for the Prevention of Cruelty to Animals. By W. H. DALRYMPLE, M.R.C.V.S. . . . . . . . . . . . . . . 21
Dairy- and Milk-Inspection. By C. COURTNEY MCLEAN, V.S. . . . . . 25
A Study of the Veterinary Surgeons' Register of Pennsylvania. By JOHN J. REPP 31

ABSTRACTS FROM FOREIGN JOURNALS:

Attempt to Exterminate Mice by Means of the Shrew-mouse Bacillus . . . 36
Regulations Against Cattle-tuberculosis in Switzerland . . . . . . . 37
Extraordinary Large Number of "Cysticercus Bovis" in a Calf Lung . . . 38
Bleeding with the Hollow Needle . . . . . . . . . . . . 38
Extirpation of Splenic Fever by Agricultural Regulations . . . . . . . 39

REPORTS OF CASES:

A Case of Spiroptera Megastoma (Rud). By S. SISSON, V.S. . . . . . . 41
Two Interesting Forms of Lameness. By W. E. A. WYMAN, V.S. . . . . . 42
Lymphadenoma, or Hodgkin's Disease. By W. HORACE HOSKINS, D.V.S. . . 43
Treatment of Purpura Hemorrhagica with Antistreptococcus Serum. By W. G. HOLLINGSWORTH, D.V.S. . . . . . . . . . . . . . . 46

EDITORIALS:

They Don't Want Our Meat-products . . . . . . . . . . 47
The Value of State Laws . . . . . . . . . . . . . . 47
Municipal Meat-inspection . . . . . . . . . . . . . 48
For Better Municipal Meat-inspection . . . . . . . . . . 48

NEW PUBLICATIONS . . . . . . . . . . . . . . . . 51
CONTROL WORK . . . . . . . . . . . . . . . . . 52
LEGISLATION . . . . . . . . . . . . . . . . . 54
GLEANINGS . . . . . . . . . . . . . . . . . . 56

SOCIETY PROCEEDINGS:

Veterinary Medical Association of New York County—Massachusetts Veterinary Association—The Missouri Valley Veterinary Medical Association—Ontario Veterinary Medical Association—The Genesee Valley Veterinary Medical Association—Veterinary Medical Society, Ontario Veterinary College—Chicago Veterinary Society—Veterinary Medical Society of the University of Pennsylvania—Keystone Veterinary Medical Association . . . . . . . 57

PERSONALS . . . . . . . . . . . . . . . . . . 64

